# TLR Agonist Immunoadjuvants Provide Effective Protection Against PCV2 and PRV Infections in a Bivalent Subunit Vaccine for PCV2 and PRV

**DOI:** 10.3390/vetsci12010025

**Published:** 2025-01-07

**Authors:** Fulai Yu, Wei Xiang, Weiye Ou, Yang Li, Xinbiao Shu, Xiaoliang Li

**Affiliations:** 1 Department of Veterinary Medicine, College of Animal Sciences, Zhejiang University, Hangzhou 310058, China; yufulai@zju.edu.cn; 2 Zhejiang Dovro Animal Health Products Co., Ltd., 298 Binhong West Road, Jinhua 321017, China; xiangwei1981213@126.com (W.X.); liyang@mebolo.com (Y.L.); 3 Zhejiang MEBOLO Biotechnology Co., Ltd., Binhong West Road, Jinhua 321017, China; ouweiye@mebolo.com; 4 Institute of Preventive Veterinary Medicine, Zhejiang Provincial Key Laboratory of Preventive Veterinary Medicine, Zhejiang University, Hangzhou 310058, China; 5 Zhejiang University-Xinchang Joint Innovation Centre (TianMu Laboratory), Gaochuang Hi-Tech Park, Xinchang 312500, China

**Keywords:** PCV2, PRV, bivalent subunit vaccine, pattern recognition receptors

## Abstract

Diseases associated with porcine circovirus type 2 (PCV2) and pseudorabies virus (PRV) significantly affect the economy of pig farms, particularly when combined infections lead to bacterial co-infections. Antigens from the pseudorabies variant strain gB and gD proteins and PCV2 (genotyped) Cap protein were mixed with the pattern recognition receptor (PRR) agonist FLICd as adjuvants and formulated with a micro-hydrogel adjuvant into PCV2 and PRV bivalent subunit vaccines. Twenty pigs, aged 30–35 days, were divided into groups A (received bivalent subunit vaccine) and B (received bivalent subunit vaccines with recombinant FLICd adjuvant), as well as C (non-vaccinated challenge control) and D (blank control). Groups A and B showed no significant difference in average daily weight gain compared to the unvaccinated controls. Fourteen days post-second vaccination, groups A and B exhibited significantly higher levels of PRV and PCV2 antibodies than groups C and D. Group B showed significantly higher average titers of PRV-specific neutralizing antibodies than group A. Fourteen days post-second vaccination, a PRV (ZJM-1 strain) challenge test was conducted. The vaccinated group achieved 100% protection. Vaccination effectively reduced virus load post-challenge and shortened the PRV shedding period. Vaccination with PCV2 and PRV bivalent subunit vaccines effectively prevents the onset of PCV2-related diseases and infections by wild pseudorabies strains.

## Simple Summary

Porcine circovirus type 2 (PCV2) is the causative agent of porcine circovirus-associated diseases (PCVADs) and immune suppression, which have been widespread in commercial farms in the swine industry worldwide. Pseudorabies virus (PRV) is the causative agent for pseudorabies, affecting swine of all ages and causing severe clinical signs, including neurological problems, fever, and respiratory distress in suckling and nursery piglets. The co-infection of pigs with PRV and PCV2, followed by secondary bacterial infections leading to porcine respiratory disease complex (PRDC) with more serious clinical symptoms and lesions, causes enormous economic losses in the pig farming industry. We developed a bivalent subunit vaccine for PCV2 and PRV with TLR agonist immunoadjuvants (FLICd), which induces high levels of PRV and PCV2 an-tibodies, providing effective protection against PCV2- and PRV-related disease until the pig fattening stage. Additionally, the bivalent subunit vaccines simplify the immunization programs by decreasing the frequency of vaccinations and reduce the likelihood of PR outbreaks caused by low antibody levels before the pigs are vaccinated by the attenuated pseudorabies vaccine.

## 1. Introduction

Porcine circovirus type 2 (PCV2) infections lead to subclinical infections and porcine circovirus-associated disease (PCAD) in pigs, which affect growth performance and cause significant economic losses to the pig industry [1]. Increasing evidence suggests that subclinical PCV2 infection results in lymphocyte depletion and the inhibition of dendritic cell activation, leading to immunosuppression [2]. Co-infection with other viruses results in severe porcine diseases. Pseudorabies (also known as Aujeszky’s disease), caused by the pseudorabies virus (PRV), causes piglet mortality, sow abortion, and respiratory diseases in fattening pigs. Since 2011, new variant strains have caused widespread PRV outbreaks in China. Co-infection with PRV and PCV2 synergistically inhibits the activation of natural immune response pathways, causing more severe immunosuppression [3], which facilitates secondary infections with pathogens, such as *Actinobacillus pleuropneumoniae* [4,5], challenging the control of respiratory infection-related diseases in intensive pig farming and resulting in more severe economic losses [6].

Currently, vaccination against PCV2 and PRV effectively reduces economic losses, owing to PCAD-related diseases and PRV outbreaks in pig herds [7]. The prevention of both diseases can be achieved by vaccination with high levels of antibodies. Therefore, vaccines developed to achieve high titers and long-lasting antibodies in pigs are effective tools for controlling diseases at different production stages. Over the past two decades, the application of immunoadjuvants has increased rapidly, driven by an enhanced understanding of the mechanisms underlying adjuvant immunoenhancement. Toll-like receptors (TLRs) in PRRs mainly include extracellular TLR1, TLR2, TLR4, TLR5, TLR6, and TLR10, as well as intracellular TLR3, TLR7, TLR8, and TLR9 [8]. TLR5 agonists, such as flagellin proteins, activate bone marrow-derived dendritic cells, enhance CD4+ helper T cell-dependent B cell humoral immune responses, and produce high levels of humoral immune responses [9]. The NIH protection experiment of co-immunization with recombinant expressed Salmonella typhi flagellin protein adjuvant and freeze-dried inactivated rabies vaccine in mice showed a significant increase in vaccine potency from 3.75 IU/mL in the blank control group to 48.69 IU/mL in the adjuvant group [10]. Therefore, in this study, antigens from the pseudorabies variant strain gB and gD proteins and the PCV2 (genotype d) Cap protein were mixed with the pattern recognition receptor (PRR) agonist FLICd as adjuvants. These were then formulated with a micro-hydrogel adjuvant into two groups of PCV2 and PRV bivalent subunit vaccines, which were administered to pigs to evaluate their effectiveness.

## 2. Materials and Methods

The study design was approved by the Laboratory Animal Welfare and Ethics Committee of Zhejiang University (ID number: ZJU20230399).

### 2.1. PRV (ZJM-1 Strain) Virus and ST Cells

ST cells were cultured in MEM supplemented with 10% newborn calf serum (provided by Inner Mongolia Opcell Biotechnology Co., Ltd., Hohhot, China) at 37 °C and 5% CO_2_. These cells, preserved by Zhejiang MEBOLO Biotechnology Co., Ltd., Jiaxing, China, were used to passage the PRV strain used in the challenge experiments and detect neutralizing antibodies in pig serum. The challenge virus was a PRV strain (ZJM-1) isolated in 2013. A sequence clustering analysis of gI/gE and gD indicated high homogeneity with the HeN1 (GenBank: KP098534.1) and TJ (GenBank: KJ789182.1) strains, classifying it as a genotype 2 variant. After plaque purification, the strain was cultured for six generations for use in the pig challenge. After being cultured for ten generations, this strain was used to challenge five piglets aged 50–60 days via intramuscular injection and nasal drip (1 mL, 10^6^ TCID_50_/mL), resulting in 3/5 fatalities and 5/5 exhibiting disease. When used to challenge five fattening pigs aged 90–100 days, the mortality and morbidity rates were 1/5 and 5/5, respectively.

### 2.2. Preparation of the PCV2 and PRV Bivalent Subunit Vaccine

Three batches of PCV2 and PRV bivalent subunit vaccines were prepared according to the detailed formulation, including the specified amounts of PCV2 and PRV antigens, adjuvants per pig dose, and the volume percentage of adjuvants used in Table 1. Pseudorabies gB and gD proteins expressed in CHO cells were purified by Ni-TED affinity chromatography (the reference sequences of the designed PRV antigens were the TJ strain variants AIT55752 and AIT55803). The recombinant PCV2b Cap protein expressed in Hi5 insect cells infected with baculovirus was purified using cation exchange chromatography. The classical carrier adjuvant, microhydrogel adjuvant 7749, was acquired from SDA Bio Co., Ltd., Sanford, NC, USA. The immunomodulating adjuvant flagellin protein (FLICd) nucleic acid, designed based on the D1 active domain of Salmonella typhimurium flic flagellin [11,12], was cloned, expressed in BL21 (DE3) Escherichia coli, and purified by nickel column chromatography after ultrasonic disruption. The vaccine was formulated by mixing the adjuvant (7749) with an aqueous phase (a solution containing PRV gB and gD proteins, PCV2d Cap protein, and immunomodulating adjuvant) in a 1:9 volume ratio. Initially, the aqueous phase was mixed and stirred at 350 rpm/min in a beaker, followed by the addition of the adjuvant (7749) to the mixture, which was stirred for an additional 30 min to produce two distinct vaccine formulations for packaging.

### 2.3. Schedule of Vaccination and Challenge

Piglets (30 to 35 days old), confirmed to be negative for nucleic acids of porcine reproductive and respiratory syndrome virus (PRRSV), African swine fever virus (ASFV), pseudorabies virus (PRV), and porcine circovirus type 2 (PCV2), were randomly allocated to four groups. Group A received the first bivalent subunit vaccine, group B received the second bivalent subunit vaccine, which included the recombinant FLICd adjuvant, group C was the non-vaccinated challenge control, and group D was the blank control group. The pigs in groups A, B, and C were challenged on day 35 post-vaccination with the PRV (ZJM-1) strain (10^6^ TCID_50_/mL) with a 1 mL nasal drip and 1 mL intramuscular injection in the neck. Serum samples were obtained prior to the primary vaccination (specifically on day 0) and on days 21, 35, and 50 post-vaccination. Subsequently, whole blood, throat swabs, and anal swabs were collected on days 3, 7, 11, and 15 post-challenge (detailed in Table 2). The timeline presentation was shown as below to provide better clarity.


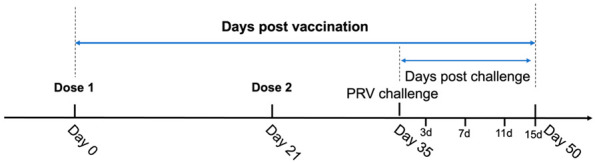


**Table 2:** Sampling schedule post-vaccination and post-challenge.

Groups	Vaccine Groups	Vaccine Dosage (IM)	Serum Sample	Whole Blood, Throat Swabs, and Anal Swabs
			Days Post-Vaccination	Days Post-Challenge
A	1	1 mL	0d/21d/35d/50d	3d/7d/11d/15d
B	2	1 mL	0d/21d/35d/50d	3d/7d/11d/15d
C	none	None	0d/21d/35d/50d	3d/7d/11d/15d
D	none	None	0d/21d/35d/50d	3d/7d/11d/15d

Note: “none” indicates that no action was performed.

### 2.4. Detection of Antibodies in Serum Samples

Serum samples were collected according to the study protocol and tested using commercial ELISA kits for PCV2-specific IgM and IgG antibodies (Ingenasa, Madrid, Spain) and PRV-specific gB and gI antibodies (kits were obtained from BioChek, Reeuwijk, the Netherlands). Testing was conducted according to the procedures outlined in the manufacturer’s manual. In the PCV2-specific antibody ELISA test setup, the critical threshold for determining positivity was set at the average OD value multiplied by 0.4 and the average OD value of the IgG positive control multiplied by 0.3. Samples exhibiting IgM and IgG antibody OD values above these critical thresholds were classified as positive, suggesting either immunization or infection in pigs. For the PRV-specific gB antibody ELISA assessment, an S/P value exceeding 0.5 indicated a positive result, whereas an S/P value of ≤0.499 signified a negative outcome. In the PRV-specific gI antibody blocking ELISA evaluation, samples with S/N values ˃ 0.55 were considered negative and those with S/N values of ≤0.55 were considered positive.

### 2.5. PRV Neutralization Assay

Pig serum samples were heat-inactivated at 56 °C for 30 min, then diluted in a 2-fold series and thoroughly mixed with the PRV (ZJM-1 strain, 6th generation) at a concentration of 100 TCID_50_/well. The mixture was incubated at 37 °C for 1 h. The neutralized mixtures (100 uL/well) were then transferred into wells of a 96-well plate, where ST cells had been grown to a monolayer, and each dilution was repeated four times. The virus-positive control wells received an equal volume of PRV without serum, whereas the negative control wells received an equal volume of MEM containing 2% NBS. The plate was incubated at 37 °C in a 5% CO_2_ atmosphere, and cytopathic effects (CPEs) were observed for 5 days. The half-protective dose (PD50) was calculated using the Reed–Muench method to determine neutralizing antibody titers. Additionally, the CPE of the virus-positive control well was calculated using the Reed–Muench method, and virus titers ranging between 100 and 200 TCID_50_/0.1 mL were considered valid.

### 2.6. Clinical Observations Post-PRV Challenge

Groups A and B were vaccinated with the PCV2 and PRV bivalent subunit vaccine, and group C, the unvaccinated control, was challenged with PRV (ZJM-1 strain) (1 mL nasal drip and 1 mL intramuscular injection) on day 35 post-vaccination. Rectal temperatures were systematically recorded, and clinical signs in all pigs were observed during the post-challenge period. Weight was recorded before the challenge and necropsy to determine the average daily weight gain.

### 2.7. Pathological Evaluation

All pigs were euthanized for tissue collection from the brain, lung lobules, and inguinal lymph nodes on day 15 post-challenge. Samples were fixed in 4% paraformaldehyde for 24 h and processed using a series of dehydration, clearing, and paraffin embedding steps. Sections were cut to a thickness of 4 µm and stained with hematoxylin and eosin (HE), following the protocol provided by the reagent kits (Shenzhen Dakewe Biotech Co., Ltd., Shenzhen, China), and sections were examined under a microscope (100× magnification) after being sealed with neutral resin (Beijing Solarbio Science & Technology Co., Ltd., Beijing, China).

ImageView 1.1 software was employed to measure lung interstitial width at 100× magnification for quantitative analysis. Briefly, each group contains 5 pigs; after measuring 3 times for each pig, the mean value of the lung interstitial width for each pig and for each group was calculated.

### 2.8. Quantification of PRV Load

On days 3, 7, 11, and 15 post-challenge, blood, oral, and anal swab samples were collected from the pigs. DNA was extracted using the EasyPure^®^ Blood Genomic DNA Kit (Beijing TransGen Biotech, Beijing, China) according to the manufacturer’s instructions. The samples were then subjected to qPCR to amplify the PRV gE gene fragment, and the resulting fluorescence was quantified against a predefined standard curve. The primers used included PRV-F 5′-GCTGTACGTGCTCGTGAT-3′, PRV-R 5′-TCAGCTCCTTGATGACCGTGA-3′, and the probe 5′-HEX-CACAACGGCCACGTCGCCACCTG-BHQ1-3′. The reaction mixture was prepared in a total volume of 25 μL, incorporating various components such as 1 μL dNTPs(10 mM), 1 μL former and reverse primers (10 mM), respectively, 0.6 μL probe (10 mM), 1.5 μL Taq enzyme(5 U/μL), 2.5 μL 10 × buffer, 2 μL template DNA, and 15.4 μL water. Cycling conditions involved initial steps at 50 °C for 2 min and 95 °C for 2 min, followed by denaturation at 95 °C for 15 s, annealing/extension and fluorescence collection at 60 °C for 30 s over 40 cycles, and a final cooling step at 25 °C for 10 s. The Applied Biosystems StepOne real-time PCR system was used for amplification and quantification, with samples considered positive if the HEX channel Ct value was ≤40 and the amplification curve exhibited a typical S-shape; samples were deemed negative if the Ct value was ˃40. A standard curve was established using PRV (ZJM-1 strain) with a titer of 10^8.0^ TCID_50_/mL, allowing for the conversion of Ct values into virus titers (half-tissue culture infective doses) based on this curve.

### 2.9. Statistical Analysis

The experimental data are presented as the mean ± standard deviation (SD) of replicates. Data were analyzed using JASP software (Team, 2020, JASP, Version 0.14.1) and Excel software using a one-way ANOVA (Levene’s test for homogeneity of variances, Bonferroni method for multiple comparison tests). The level of significance was set at *p* < 0.05.

## 3. Results

### 3.1. Safety Assessment of the PRV and PCV2 Bivalent Subunit Vaccine

**Figure 1:**
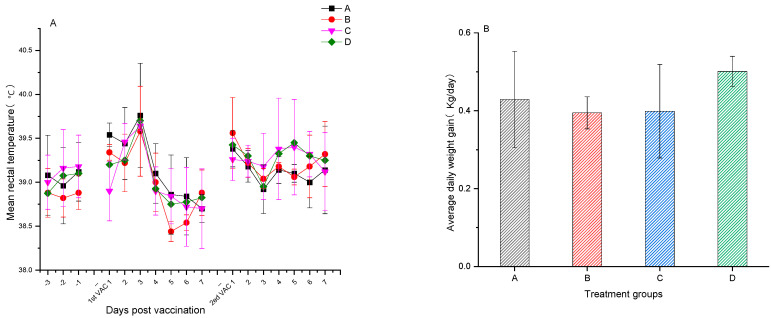
(**A**) Average rectal temperatures ± SD of experimental pigs in each group three days before vaccination and seven days after the primary and second booster vaccination. (**B**) Average daily weight gain ± SD of experimental pigs in each group from the primary vaccination to 14 days after the second booster vaccination.

Rectal temperatures were recorded for the experimental pigs three days before the initial immunization and seven days following the first and second immunizations, with temperatures ranging between 38.5 and 39.5 °C. No significant difference was observed in rectal temperatures between the immunized groups A and B and the unimmunized control groups C and D. Similarly, no significant temperature variation was noted within the immunized groups before and after vaccination (Figure 1A). All pigs maintained a rectal temperature below 40.0 °C throughout the experiment, with no reduction in feed intake, noticeable swelling, or other adverse effects at the injection sites. On day 0 of primary vaccination and day 14 post-second booster, all pigs were weighed, and the average daily weight gain for groups A, B, C, and D was 0.43 ± 0.12, 0.39 ± 0.04, 0.40 ± 0.12, and 0.50 ± 0.04, respectively. No significant difference was observed in the average daily weight gain among the groups (*p* > 0.05) (Figure 1B), indicating the safety of PCV2 and PRV bivalent subunit vaccines.

### 3.2. Results of ELISA Antibody Tests Post-Immunization with the PCV2 and PRV Bivalent Subunit Vaccine in Experimental Pigs

**Figure 2:**
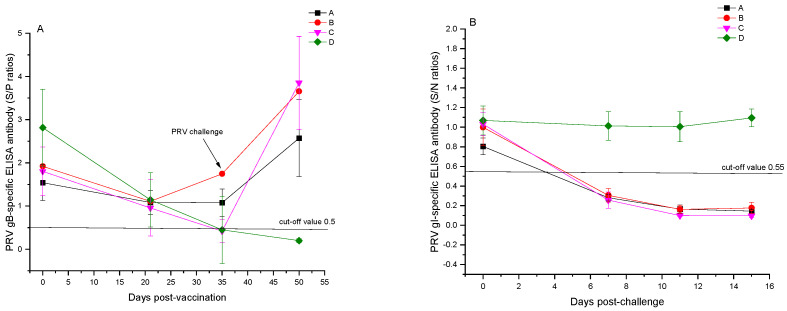
ELISA detection results for PRV-specific antibodies. (**A**) PRV gB-specific antibody ELISA detection results from pre-vaccination to 15 days post-challenge in experimental pigs from each group. (**B**) PRV gI-specific antibody ELISA detection results before and after challenge in experimental pigs from each group.

All 20 experimental pigs tested positive for maternal antibodies against PRV, with a 100% positivity rate for PRV gB-specific ELISA antibodies prior to immunization and the lowest S/P value of 1.5. Fourteen days following the booster vaccination, the average S/P values for PRV-specific gB protein ELISA antibodies in groups A and B were 1.07 ± 0.32 and 1.74 ± 0.83, respectively, showing no significant variance among the immunization groups (*p* > 0.05), all with a 5/5 positivity rate. The mean S/P values for gB protein ELISA antibodies in the unvaccinated control groups C and D were 0.42 ± 0.27 and 0.45 ± 0.78, with positivity rates of 1/5 and 2/5, respectively. Immunization with the bivalent subunit vaccine effectively generated PRV gB-specific ELISA antibodies in piglets carrying maternal antibodies (Figure 2A). Prior to the viral challenge, all groups were negative for PRV gI-specific ELISA antibodies, suggesting that the experimental pigs were not exposed to the wild-type PRV strain (Figure 2B).

Fifteen days post-challenge, the S/P values for PRV gB-specific ELISA antibodies in the vaccinated groups A and B and the unvaccinated control group C increased significantly to 2.57 ± 0.89, 3.65 ± 0.96, and 3.85 ± 1.07, respectively, with no significant difference in the average S/P values among the challenged groups (*p* > 0.05). The S/P value for the blank control group D remained at 0.20 ± 0.05, continuing to test negative in 5/5 cases. Seven days post-challenge, the S/N values for PRV gI-specific ELISA antibodies in all challenged groups were 0.28 ± 0.03, 0.31 ± 0.07, and 0.26 ± 0.08, respectively. The challenged control group C showed the lowest S/N value; however, no significant difference was observed compared to groups A and B (*p* > 0.05), with all challenged groups turning positive. For the blank control group D, the S/N values for PRV gI-specific ELISA antibody tests were all above 0.55, testing negative in 5/5 cases during the study period (Figure 2B), indicating no PRV infection.

**Figure 3:**
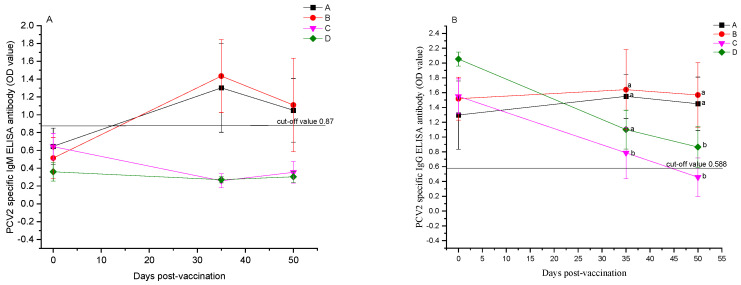
Outcomes of ELISA tests for PCV2-specific antibodies among various experimental pig groups. (**A**) Results of PCV2-specific IgM antibody ELISA tests conducted on different experimental pig groups prior to immunization and 14 and 29 days following the second vaccination. (**B**) Results of PCV2-specific IgG antibody ELISA tests conducted on different experimental pig groups prior to vaccination and 14 and 29 days following the second vaccination. The data were analyzed by one-way ANOVA, and alphabet letters (a and b) different between groups represent statistically significant difference.

Before vaccination, the experimental pigs tested positive for PCV2-specific IgG antibodies and negative for IgM via the ELISA, indicating the presence of maternal antibodies and no infection with PCV2. Fourteen days following the booster vaccination, pigs in the PCV2 and PRV bivalent subunit vaccinated groups A and B exhibited PCV2-specific IgM antibody ELISA OD values of 1.3 ± 0.5 and 1.09 ± 0.26, respectively, all converting to positive (exceeding the standard threshold of 0.87). Group B had the highest mean OD value; however, no significant difference was observed in the mean OD values among the vaccinated groups (*p* > 0.05). Pigs in the non-vaccinated groups C and D had PCV2-specific IgM antibodies at 0.35 ± 0.12 and 0.30 ± 0.06, respectively, both remaining negative, indicating a robust immune response in groups A and B post-vaccination. Control groups C and D continued to test negative for IgM 29 days after the booster shot, demonstrating that the experimental pigs did not contract PCV2 during the study period (Figure 3A). Owing to elevated maternal antibody levels, pigs in group B had an average OD value of 1.64 ± 0.54 for PCV2-specific ELISA IgG antibodies 14 days post-booster, higher than those in groups A with 1.54 ± 0.30, with no significant differences between the groups (*p* > 0.05). The OD values for PCV2-specific IgG antibodies in control groups C and D were 0.79 ± 0.35 and 1.10 ± 0.26, respectively, with a notable distinction observed between the vaccinated groups and control group C (*p* < 0.05), whereas pigs in group D maintained high titers of maternal antibodies. Twenty-nine days following the second vaccination (50 days post-initial vaccination), the vaccinated groups A and B had average OD values for PCV2-specific IgG ELISA antibodies at 1.45 ± 0.36 and 1.57 ± 0.43, all testing positive in 5/5 cases, exceeding the unvaccinated control groups D and E with average OD values of 0.46 ± 0.26 and 0.87 ± 0.28, with positivity rates of 2/5 and 5/5, respectively (*p* < 0.05, Figure 3B). Vaccination with PCV2 and PRV bivalent subunit vaccines in piglets with high maternal antibodies resulted in the maintenance of elevated antibody levels in piglets aged 70–80 days.

### 3.3. Results of PRV-Neutralizing Antibody Tests

**Figure 4:**
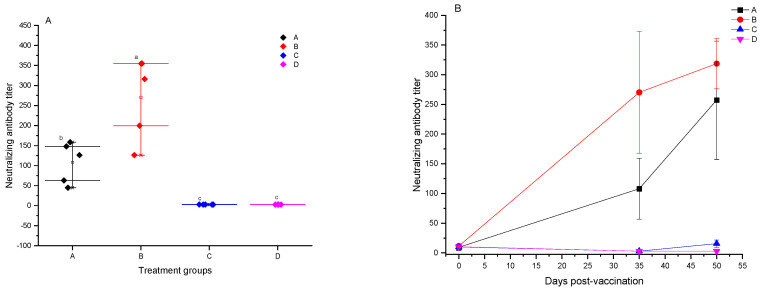
Results of testing for PRV-specific neutralizing antibodies across various experimental pig groups. (**A**) The average value ± SD of PRV-specific neutralizing antibodies measured 14 days following the second vaccination in different groups of experimental pigs. The data were analyzed by one-way ANOVA, and alphabet letters (a, b, and c) different between groups represent statistically significant difference. (**B**) The average value ± SD of PRV-specific neutralizing antibodies measured prior to vaccination, 14 days after the second vaccination, and 15 days post-challenge in different groups of experimental pigs.

Experimental pigs with maternal antibodies against PRV showed neutralizing antibody titers of 1:9.7 ± 3.9. Pigs in group B, vaccinated with the FLICd adjuvant-enhanced vaccine, exhibited PRV (ZJM-1 strain)-specific neutralizing antibody titers of 1:270.2 ± 102.8 14 days post-booster, significantly outperforming the adjuvant-free group A with titers of 1:108 ± 51.2 (*p* < 0.05), thus effectively boosting PRV-neutralizing antibody levels. In contrast, non-vaccinated control groups D and E tested negative for neutralizing antibodies (<1:4). PCV2 and PRV bivalent subunit vaccines successfully induced the production of PRV-specific neutralizing antibodies in vaccinated pigs. Fifteen days following the PRV exposure, the average titers of neutralizing antibodies in groups A and B increased to 257.2 ± 99.6 and 318.6 ± 42.3, respectively, whereas unvaccinated control group C was 1:15.4 ± 6.4 (Figure 4B). The observed trends in neutralizing antibody responses were similar to those observed in the PRV-specific gB antibody ELISA tests, although without a direct linear relationship between the actual figures. This indicates that neutralizing antibody testing in vaccine evaluations should not rely solely on the ELISA method.

### 3.4. PRV Challenge of Pigs

**Figure 5:**
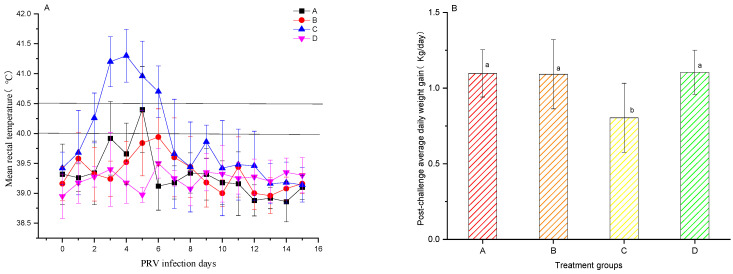
(**A**) Average rectal temperatures ± SD for various experimental pig groups at 15 days following exposure to the challenge agent. (**B**) Average daily weight gain ± SD for various experimental pig groups at 15 days post-challenge. The data were analyzed by one-way ANOVA, and alphabet letters (a and b) different between groups represent statistically significant difference.

Groups A and B comprised pigs vaccinated with the PCV2 and PRV bivalent subunit vaccines, and the unvaccinated group C was challenged with PRV (ZJM-1 strain). Rectal temperatures were meticulously recorded for 15 days post-challenge (Figure 5A). On day 3 post-challenge, all pigs in the unvaccinated control group C experienced body temperatures exceeding 41 °C, with temperatures above 40.5 °C persisting for 4 days. In contrast, in groups A and B, the temperatures in individual pigs exceeded 40 °C starting on day 3, though this elevation lasted for less than 48 h. Between days 2 and 6 post-challenge, the average rectal temperatures in groups A and B were significantly lower than those in the unvaccinated control group C (*p* < 0.05). Weigh-ins conducted before the challenge and 15 days afterward revealed that the average daily weight gains in the vaccinated groups A and B and the blank control group D were 1.10 ± 0.16, 1.09 ± 0.23, and 1.11 ± 0.15 kg, respectively, showing no significant variance among the groups. The unvaccinated challenge control group C had a notably lower average daily gain of 0.80 ± 0.23 kg, significantly lower than groups A, B, and D (*p* < 0.05, Figure 5). Vaccination effectively safeguards against PRV, mitigating the economic impacts associated with reduced daily weight gain owing to sickness.

### 3.5. Detection of Viral Load Post-PRV Challenge

**Figure 6:**
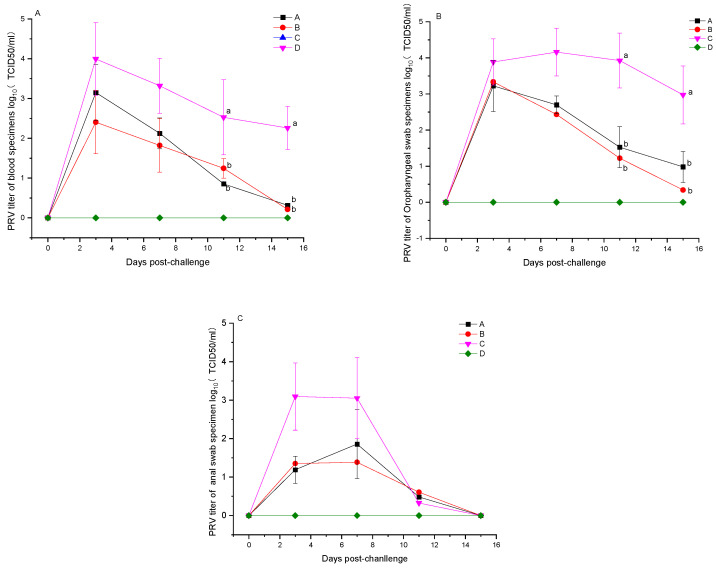
Viral load testing results for blood, throat swab, and anal swab samples from experimental pigs in groups A, B, C, and D before the PRV challenge and 15 days afterward for the five tests. (**A**) Average PRV viral loads ± SD in five whole blood samples from pigs for the four groups. (**B**) Average PRV viral loads ± SD in five throat swab samples from pigs for the four groups. (**C**) Average PRV viral loads ± SD in five anal swab samples from pigs for the four groups. For A and B, the data were analyzed by one-way ANOVA, and alphabet letters (a and b) different between groups represent statistically significant difference.

Following the challenge with PRV, the experimental pigs had their blood, throat swabs, and anal swabs collected as scheduled, and the viral load was determined by quantitative RT-PCR. All samples from the blank control group D tested negative across the board (5/5). On day 3 post-challenge, the whole blood samples from groups A, B, and C exhibited viral loads (log10TCID_50_/mL) of 3.15 ± 0.70, 2.41 ± 0.79, and 4.00 ± 0.91, respectively, all testing positive, with group C showing a viral load higher than A and B (*p* > 0.05). By day 11 post-challenge, the viral loads (log10TCID_50_/mL) in the blood samples of the vaccinated groups A and B were 0.85 ± 0.05 and 1.24 ± 0.25, respectively, significantly lower than the 2.53 ± 0.94 observed in group C (*p* < 0.05). Fifteen days post-challenge, the vaccinated groups A and B showed average viral loads (log10TCID_50_/mL) in their blood samples of 0.31 and 0.21, respectively, with the challenged control group C recording an average viral load of 2.26 ± 0.54, demonstrating significantly lower levels in the vaccinated groups compared to the control (*p* < 0.05) (Figure 6A); the turning negative rates for the vaccinated groups A and B were 4/5 and 4/5, respectively, compared to 0/5 in the challenge control group C. The viral loads detected in throat swab samples showed a similar trend to those in blood samples, with significantly lower levels in vaccinated groups A and B than in group C on days 11 and 15 post-challenge (*p* < 0.05) (Figure 6B). By day 15 post-challenge, the ratio of throat swab samples turning negative was 2/5 and 4/5 for groups A and B, respectively, exceeding the 0/5 in group C. On day 3 post-challenge, the anal swab samples from groups A and B had average viral loads (log10TCID_50_/mL) of 1.18 ± 0.35 and 1.35 ± 0.31, respectively, lower than group C’s 3.09 ± 0.87 (*p* > 0.05). By day 11 post-challenge, the proportions of anal swab samples that turned negative were 4/5, 3/5, and 3/5 in groups A, B, and C, respectively. On day 15 post-challenge, all anal swab samples from the challenged pigs tested negative (Figure 6C). These findings demonstrate that immunization with the PCV2 and PRV bivalent subunit vaccines, in comparison to the non-vaccinated group C, effectively reduced the PRV load in the blood and excretion of pigs in groups A and B, thereby shortening the duration of viral shedding post-infection.

### 3.6. Post-PRV Challenge Clinical Signs and Pathological Examination

Fifteen days after the PRV challenge, clinical observations were made regarding temperature fluctuations and respiratory and neurological symptoms, including a compilation of disease incidence data for pigs in groups A, B, C, and D (Table 3). All pigs in the unvaccinated challenge control group C (5/5) exhibited disease symptoms, whereas none (0/5) in the vaccinated groups A and B showed any signs of illness. When increased to 70–80 days of age, pigs in group C, challenged with the genotype 2 variant strain of PRV (ZJM-1), developed signs indicative of interstitial pneumonia and exhibited high fever, poor mental condition, and, in some cases, tremors and neurological symptoms. These pigs also exhibited food intake decreases and impaired weight gain. However, pigs that were not affected by the disease exhibited a resolution of their clinical signs and regained their normal health. Conversely, pigs in the vaccinated groups A and B exhibited no significant clinical signs of pseudorabies infection.

**Figure 7:**
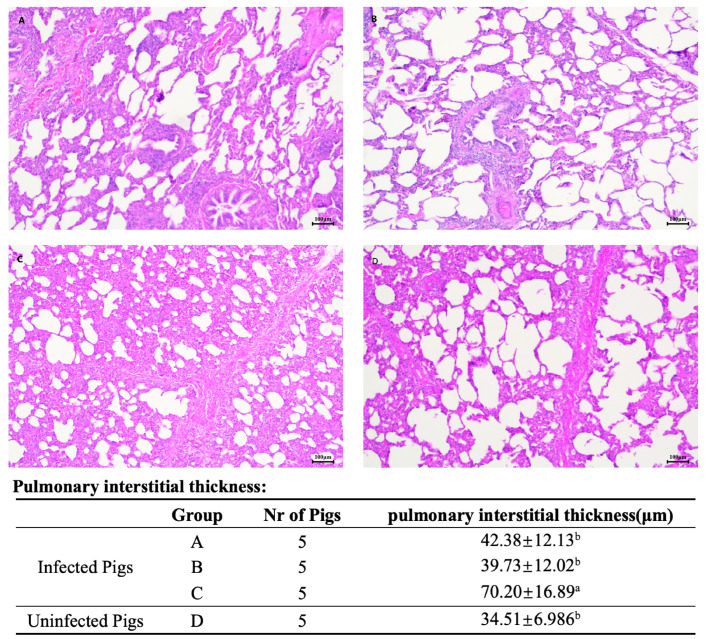
Hematoxylin- and eosin-stained lung tissue sections from groups (**A**–**D**) of experimental pigs, 15 days post-PRV challenge. The lung sections are magnified by 100× and are represented as images (**A**–**D**) in the figure, representing groups (**A**–**D**). Compared to the blank control group (**D**), the lung tissues of the vaccinated groups (**A**,**B**) show a slight increase in the width of the alveolar interstitium. The most severe interstitial widening was observed in the unvaccinated group (**C**). The data were analyzed by one-way ANOVA, and alphabet letters (a and b) different between groups represent statistically significant difference.

By day 15 post-challenge, all pigs in the challenge groups had their clinical signs resolved. A comprehensive pathological examination revealed no significant lesions in the lungs, brain, spleen, or inguinal lymph nodes of any of the experimental pigs. Tissues from the lungs, brain, and inguinal lymph nodes were fixed and processed for hematoxylin- and eosin-stained histological examination. The examination of brain and inguinal lymph node sections from all pigs in the challenge groups showed no significant signs of inflammatory cell infiltration or normal tissue structures. Lung sections from all pigs in the challenge groups exhibited varying degrees of alveolar interstitial widening compared with the blank control group D. Notably, the unvaccinated challenge group C exhibited more severe alveolar interstitial widening and more pronounced interstitial pneumonia than the vaccinated challenge groups A and B (HE staining at 100× magnification, Figure 7).

## 4. Discussion

**Figure 8:**
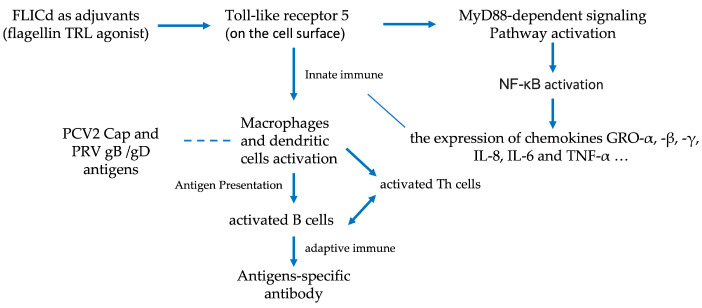
The recombinant flagellin TLR agonist FLICd binds to TLR5 on the surface of different cell types (macrophages, dendritic cells, lymphocytes, etc.). By activating the MyD88-dependent signaling pathway and the NF-κB signaling pathway, FLICd induces the expression of a series of cytokines, such as IL-6 and IL-8, and activates the innate immune cells. The activated macrophages and dendritic cells present vaccine PCV2 and PRV antigen proteins to B cells and helper somatic cells (CD4) Th cells, further activating effector B cells to express antibodies to PCV2- and PRV-specific antigens.

In the context of DNA viral infections, immune cells can recognize viral DNA through pattern recognition receptors, such as Toll-like receptor 9 (TLR9), cyclic GMP-AMP synthase (cGAS), and the DNA-dependent activator of IRFs (DAI). These receptors initiate a cascade of signaling pathways that result in the expression of type I interferons (IFNs) and pro-inflammatory cytokines, thereby influencing a specific immune response [13]. Viral proteins interact with host cell molecules; for example, PRV proteins like UL13, UL24, UL42 [14], and gE inhibit the NF-kB and cGAS-STING signaling pathways, suppress the innate immune response, and promote an environment conducive to viral replication within infected pig cells. Although no direct correlation exists between the strength of innate immune responses and the magnitude of antigen-specific immune responses, activating innate immunity to a certain threshold is essential for enhancing effective antigen-specific immune responses. Compared with the intricate immune responses triggered by viral infections, subunit antigens have a relatively limited capacity to activate innate immune responses. Immunomodulating adjuvants such as FLICd can activate dendritic cells, thereby enhancing specific B cell-mediated humoral immune responses [9,11]. Vaccination of groups A and B with PCV2 and PRV bivalent subunit vaccines demonstrated that pigs in group B, vaccinated with the FLICd adjuvant-enhanced vaccine, exhibited significantly higher mean neutralizing antibody titers against PRV (ZJM-1 strain) on day 14 post-second vaccination than those in group A (*p* < 0.05). Group B showed higher mean ELISA OD values for PCV2-specific IgG antibodies than group A, although the difference was not statistically significant (*p* > 0.05). This study indicates that FLICd can enhance antibody levels in vaccinated pigs and is a potential immunomodulatory adjuvant for subunit vaccines (Figure 8).

Rectal temperatures were recorded for seven days before primary vaccination and after vaccination with the PCV2 and PRV bivalent subunit vaccine, with no significant differences among groups A, B, C, and D (*p* > 0.05) and no cases of temperatures consistently exceeding 40 °C twice. After immunization, groups A and B exhibited no adverse reactions, such as the swelling of the injection site. Weights were measured before vaccination and on day 14 post-booster vaccination, and no significant difference was observed in the average daily weight gain between vaccinated groups A, B, C, and D (*p* > 0.05). The results showed that the PCV2 and PRV bivalent subunit vaccines were safe for pigs.

Eight genotypes of PCV2 strains [15], each with varying virulence levels, have been identified. Some strains cause high viremia and severe pathological damage [16]. Pigs were administered the PCV2 vaccine-induced neutralizing antibodies, which showed higher titers against the vaccine PCV2 strain genotype than that against other genotypes; however, no significant differences were observed, indicating cross-protection against different PCV2 genotypes [17,18]. A positive correlation was observed between the PCV2-specific IgG antibody levels, neutralizing antibody levels [19,20], and protection against PCV2 challenge [21]. Therefore, we did not perform a PCV2 challenge efficacy study. All pigs in groups A and B tested positive for PCV2-specific IgM antibodies on day 14 post-vaccination, and some pigs tested positive for IgM antibodies on day 29 post-second vaccination, indicating a robust, specific immune response to the PCV2 and PRV bivalent subunit vaccine. Although all piglets had high titer maternal antibodies, vaccinated groups A and B exhibited significantly higher PCV2-specific ELISA IgG antibodies than the unvaccinated control groups C and D (*p* < 0.05) on day 29 post-vaccination. All vaccinated piglets grown to 70–80 days of age had high titers of PCV2-specific antibodies that protected them against PCV2.

Reasonable concordance existed between PRV gB-specific antibodies detected by ELISA and PRV-neutralizing antibodies in vaccinated groups A and B, with similar trends but no linear relationship. Although there were similar average ELISA S/P values for gB-specific antibodies using ELISA kits across groups A, B, and C, the neutralizing antibody titer of group C was significantly lower than that of the vaccinated groups post-PRV challenge. These results suggest that ELISA cannot replace the evaluation of PRV-protective neutralizing antibodies. The positive rate of PRV gI-specific antibodies detected by ELISA on day 7 post-PRV challenge aligned with the rate of viral load in all samples, proving to be an effective method for evaluating wild virus infection. The challenge strain of PRV (ZJM-1), a genotype 2 variant strain identified in 2013, demonstrated a mortality rate of 3/5 and a disease rate of 5/5 when used to challenge pigs aged 50–60 days using a similar method as in this experiment. All vaccinated groups showed 0/5 disease incidence during this challenge, and the challenged control group had 5/5 disease incidence; however, all challenged pigs survived. Studies indicate that as pigs age to 5 weeks, the viral load of PRV (Kaplan strain) infection significantly decreases in the central nervous system, such as the cerebellum and thalamus [22], as well as in peripheral nerves, such as the trigeminal nerve, which may explain the mild tremor neurological symptoms exhibited by only 2/5 pigs in this study. The experimental pigs (aged 70–80 days) exhibited clinical signs but no fatalities and respiratory signs recovered on day 15 post-challenge, possibly due to the absence of a secondary infection. All challenged pigs were euthanized 15 days post-challenge and showed no significant pathological changes in organs such as the brain, lungs, liver, kidneys, or inguinal lymph nodes. Lung pathology sections (HE staining) revealed slight interstitial widening in groups A and B, with the unvaccinated challenge control group C showing more pronounced widening, indicating that severe interstitial pneumonia occurred post-challenge, and these results showed that the vaccine provided effective protection. One week post-challenge, pigs in the challenge control group C experienced high fever, poor mental state, reduced food intake, and respiratory signs such as coughing and respiratory rapidity, likely contributing to reduced weight gain. On day 3 post-challenge, the viral load in blood samples from all challenge groups peaked and gradually decreased thereafter, with the positivity rates of blood samples in groups A and B on day 15 being 1/5 and 1/5, respectively, whereas the challenge control group C remained at 5/5 positive. Vaccination with the PCV2 and PRV subunit vaccines induced high-titer PRV-specific neutralizing antibodies, reducing the viral load of PRV (ZJM-1 strain) in the blood post-challenge infection, as well as reducing the viral load spread through the mouth and feces, shortening the shedding time, effectively preventing PRV disease, and reducing pig weight loss due to disease. The emergence of PRV variants increases the pathogenic virulence in pigs and has led to diseases in pigs vaccinated with the Bartha-k61 attenuated vaccine [23,24,25]. However, vaccination with the PRV Bartha strain-attenuated vaccine can provide 60% or complete protection against PRV variants [24,26,27], which corresponds to its lower specific neutralizing antibody titers against variant strains than classical strains. The neutralizing antibody titers produced by the variant strain-attenuated vaccine against the classical PRV strain (LA strain) were similar to those of the Bartha-K61 vaccine but significantly higher against PRV variant strains than against non-variant strains (Bartha-K61, HB2000, and SA215), effectively protecting against variant and classical strains in experimental pigs [26,28]. This indicates that PRV remains a single serotype and that improving the titer and affinity of neutralizing antibodies is crucial for PRV disease prevention and epidemic control. High-titer maternal antibody piglets vaccinated with the PCV2 and PRV bivalent subunit vaccines had a 100% positive rate for PCV2- and PRV-specific antibodies compared to the unvaccinated control group. This vaccination strategy effectively avoided maternal antibody interference and minimized the risk of PCV2 and PRV infection associated with delayed vaccination schedules. PCV2 and PRV bivalent subunit vaccines are safe, can effectively prevent PCV2 and PRV diseases, and can reduce the economic loss caused by respiratory diseases due to secondary mixed bacterial infections in high-density pig farming.

## Figures and Tables

**Table 1:** The formulation of the PCV2 and PRV bivalent subunit vaccine.

Groups	Adjuvant (*v*/*v*)	Immunomodulating Adjuvant	Antigen	
			PRV	PCV2
A	7749 1/9	Non	60 μg gB 60 μg gD	70 μg
B	7749 1/9	100 μg FLICd	60 μg gB 60 μg gD	70 μg

**Table 3:** Summary of clinical signs and disease incidence of the pigs in different experimental pig groups for 15 days post-PRV challenge.

Group	Number	Virus Titer (TCID_50_/mL)	Challenge Dose (mL)/Challenge Route	Clinical Signs			Morbidity	Mortality
				Recital Temperature (>40.5 °C)	Nervous Signs	Respiratory Signs		
A	5	10^6.0^	1/IM + 1/IN	0/5	0/5	0/5	0/5	0/5
B	5	10^6.0^	1/IM + 1/IN	0/5	0/5	0/5	0/5	0/5
C	5	10^6.0^	1/IM + 1/IN	5/5	2/5	5/5	5/5	0/5
D	5	None	None	0/5	0/5	0/5	0/5	0/5

## Data Availability

The raw data supporting the conclusions of this article will be made available by the authors upon request.
